# Volumetric modulated arc therapy is superior to conventional intensity modulated radiotherapy - a comparison among prostate cancer patients treated in an Australian centre

**DOI:** 10.1186/1748-717X-6-108

**Published:** 2011-09-05

**Authors:** Gerald B Fogarty, Diana Ng, Guilin Liu, Lauren E Haydu, Nastik Bhandari

**Affiliations:** 1 Radiation Oncology Department, Mater Hospital, Crows Nest, NSW, Australia; 2 Research and Biostatistics, Melanoma Institute Australia, North Sydney, NSW, Australia; 3 Sydney Medical School, the University of Sydney, Sydney, NSW, Australia

**Keywords:** Intensity-modulated radiotherapy, Volumetric modulated arc therapy, three-dimensional conformal radiotherapy, Australia, health care cost

## Abstract

**Background:**

Radiotherapy technology is expanding rapidly. Volumetric Modulated Arc Therapy (VMAT) technologies such as RapidArc^® ^(RA) may be a more efficient way of delivering intensity-modulated radiotherapy-like (IM) treatments. This study is an audit of the RA experience in an Australian department with a planning and economic comparison to IM.

**Methods:**

30 consecutive prostate cancer patients treated radically with RA were analyzed. Eight RA patients treated definitively were then completely re-planned with 3D conformal radiotherapy (3D); and a conventional sliding window IM technique; and a new RA plan. The acceptable plans and their treatment times were compared and analyzed for any significant difference. Differences in staff costs of treatment were computed and analyzed.

**Results:**

Thirty patients had been treated to date with eight being treated definitely to at least 74 Gy, nine post high dose brachytherapy (HDR) to 50.4Gy and 13 post prostatectomy to at least 64Gy. All radiotherapy courses were completed with no breaks. Acute rectal toxicity by the RTOG criteria was acceptable with 22 having no toxicity, seven with grade 1 and one had grade 2.

Of the eight re-planned patients, none of the 3D (three-dimensional conformal radiotherapy) plans were acceptable based on local guidelines for dose to organs at risk. There was no statistically significant difference in planning times between IM and RA (p = 0.792). IM had significantly greater MUs per fraction (1813.9 vs 590.2 p < 0.001), total beam time per course (5.2 vs 3.1 hours, p = 0.001) and average treatment staff cost per patient radiotherapy course ($AUD489.91 vs $AUD315.66, p = 0.001). The mean saving in treatment staff cost for RA treatment was $AUD174.25 per patient.

**Conclusions:**

3D was incapable of covering a modern radiotherapy volume for the radical treatment of prostate cancer. These volumes can be treated via conventional IM and RA. RA was significantly more efficient, safe and cost effective than IM. VMAT technologies are a superior way of delivering IM-like treatments.

## Background

Radiotherapy technology is expanding rapidly. Newer technologies such as intensity modulated radiotherapy (IM) enable better radiation dose conformality to the target volume compared with three-dimensional conformal radiotherapy (3D). Better dose conformality means that the dose of radiation to the volume requiring treatment can be escalated, thereby increasing cancer control, more volume can also be treated safely, while simultaneously decreasing the dose to surrounding radiation-sensitive normal tissues, thereby decreasing radiotherapy toxicities.

These technologies have been slow to be embraced in the Australian setting compared to other developed countries for various reasons. For example, IM has been a standard therapy in the United States from mid-1995, whereas in Australia it is still not offered in every department and even then is reserved for special situations, for example, radical re-treatments and paediatric cases. However, there have been recent developments at a governmental level to investigate whether conventional IM has benefits over 3-DCRT. This project will ensure that increased government reimbursement for therapies is based on proper evidence. This process has been followed before with success [1].

In the meantime, IM technology has evolved even further. IM technology can now be delivered in a more efficient manner via Volumetric Modulated Arc Therapy (VMAT). VMAT technologies may also be safer. External beam radiotherapy is delivered by a certain number of machine monitor units (MUs), a measure of machine radiation output. MUs are important as second cancer risk in patients treated with radiotherapy is proportional to how many MUs are needed per treatment course [2,3].

The efficiency of VMAT has enabled the expansion of IMRT-like techniques to routine treatments and not rationed to only rare situations. The Mater Hospital in Sydney was the first centre in the state of New South Wales of Australia to treat with VMAT. This was possible following the installation of a new Varian^® ^21iX linear accelerator, which delivers VMAT under the trade name of RapidArc^® ^(RA). The department went directly from treating with 3D to RA. Over 350 patients have now been treated with this new technology in this centre. This study is an audit of the RA experience to ensure that the newer therapy is recommended with proper evidence.

## Methods

The audit is of the first thirty consecutive prostate cancer patients that have been radically treated for prostate cancer by one radiation oncologist with RA.

Data gathered for these prostate cancer patients included the indication for radiation therapy. This was either definitive radiation (74 to 78 Gy); or post surgery radiation (64 to 66 Gy); or post high dose rate brachytherapy (50.4 Gy). Also collected were total beam time, total MUs per course and acute rectal toxicities as per the RTOG criteria [4] up to six weeks post radiotherapy.

Plans were accepted by the treating radiation oncologist if the IM dose constraints for external beam radiotherapy for prostate cancer were met as per the current local guidelines as detailed in Table 1. These constraints are essentially from Emani et al [5].

**Table 1:** Rectal dose constraints for 3D and IM as per local guidelines [5]

Dose (Gy)	% of total Rectum receiving dose
40Gy	≤ 60% (≤35% with IM)

65Gy	≤ 40% (≤17% with IM)

70Gy	≤ 30%

75Gy	≤ 10%

### Comparison with replanning

The RA patients treated with definitive external beam radiotherapy were then completely re-planned with 3D; and a conventional sliding window IM technique; and a new RA plan. The IM technique was with a seven field plan, RA was planned using two arcs. Planning was done by a dosimetrist, qualified and experienced in this type of planning, but not familiar with these particular cases. The planning system used was Eclipse^® ^version 8.6 and was imported into the treatment system using Mosaiq^® ^version 2.00W9. The time taken for the dosimetrist to plan for each of the techniques for each case was recorded. Quality assurance on a phantom was then performed by a qualified physicist as per local protocol using our in-house phantom. It was assumed that there was no need for a quality assurance of 3D.

The phantom was then treated. The default dose rate used was 600MUs per minute. MUs per fraction were recorded. The beam time from start to finish of each fraction for all acceptable techniques was recorded. The total beam time for each technique was computed by multiplying this time by the number of fractions. The number of fractions prescribed was the same for the patient independent of technique. These data were then compared and analyzed for any significant difference.

### Comparison of treatment cost between IM and RA

Economic remuneration data for treatment radiation therapists was gleaned from the current New South Wales (NSW) award [6] (Table 2). This information on payment per hour allowed an item for treatment staff costs to be estimated. Labour costs were computed for the total course. It was assumed that the treating radiation therapists were on the lowest paid level qualified to perform the relevant duties. In our NSW system, this meant that the two therapists involved in the treatment, were level 4, grade 1, year 1; and level 2, year 1 respectively. The difference in cost between the techniques was computed by multiplying the total treatment beam times by the staff remuneration per hour. In arriving at the different costs, it was assumed that the only difference between the treatments was the total duration of beam-on time from start to finish of each fraction. It was assumed that patient setup time and position verification, usually with an IGRT (image guided radiotherapy) technique, was the same independent of technique. The treatment costs of each technique were compared and analyzed for any significant difference.

**Table 2:** Costs of radiotherapy staff in the planning and treatment of cancer patients as per the New South Wales award of 2011 [6].

Position	$AUD/hr
Treating Radiation therapist - level 4, grade 1, year 1	$53.43

Treating Radiation therapist - level 2, year 1	$29.37

Total labour cost of treating team	$82.80

It was assumed that the following were the same independent of the technique: time spent by the radiation oncologist to perform contouring, plan acceptance and to see the patients in follow up; the costs of the different linear accelerators (as all new modern linear accelerators are now capable of 3D, IM and VMAT); and costs of linear accelerator commissioning by physics for the different techniques. The latter are not considered important between the techniques as commissioning is a one off cost for these machines which have a working life of around 10 years.

Statistical analyses were conducted using the IBM SPSS Statistic 19.0 software package. Independent and paired t-tests were used to compare mean values where appropriate. Two-tailed p-values < 0.05 were considered statistically significant.

## Results

Thirty consecutive prostate cancer patients treated radically via RA by one radiation oncologist in our institution were found and their characteristics are detailed in Table 3.

**Table 3:** Indication for radiotherapy, total beam times, monitor units and acute rectal toxicity of the first 30 prostate patients treated with RA.

Indication For Radiation	No Of Patients	Total Beam time (minutes)	Total Monitor Units	Acute Bowel Toxicity (RTOG Grade)
Definitive	8	186	23050	4 × grade 1, 1 × grade 2

Post HDR	9	132	16496	2 × grade 1

Post surgery	13	138	21136	1 × grade 1

Eight of these RA patients, those treated with definitive external beam radiotherapy were re-planned with 3D, conventional IM and RA techniques. None of the 3D plans that were attempted were acceptable by the local guidelines as per Table 1 for the 3D criteria. All the RA and IM plans were acceptable according to PTV coverage and the dose constraints for IM as detailed in Table 1. Planning times between IM and RA (Table 4) were not significantly different (p = 0.792). There was significantly greater machine output (MUs) per fraction for IM (1813.9, SD = 159.1) compared with RA (590.2, SD = 67.1); p < 0.001.

**Table 4:** IM and RA planning, and Monitor units

Patient number	Planning Time (mins) p = 0.792		MU's/fraction p < 0.001	
	
	*RA*	*IM *	*RA*	*IM*
1	73	79	688	1589

2	75	82	589	1909

3	81	61	588	2025

4	85	74	506	1656

5	84	65	625	1898

6	48	72	544	1646

7	80	99	667	1889

8	87	67	515	1899

Total treatment times (hours) were significantly greater for IM (5.2, SD = 1.2) compared with RA (3.1, SD = 0.5); p = 0.001 as detailed in Table 5. This table also records the cost difference between the techniques using the data of Table 2. The average cost per patient for IM treatment ($ AUD 489.91, SD = $ AUD 107.53) was significantly higher than that of RA ($AUD315.66, SD = $ AUD 51.59), p = 0.001. The mean saving in cost for RA treatment was $ AUD 174.25 per patient (95% CI: $95.38-$253.11). This cost saving is only for the wages of the treating radiation therapy staff, based on the difference in the beam times between RA and IM. The analyzed data of IM versus RA is summarized in Table 6.

**Table 5:** IM and RA treatment times and relative treatment staff costs

Pt No	Dose(Gy)/fraction	Total Treatment Beam Time (hours)		Difference in treatment (Time: hr/min; Cost: $)
			
		*IM*	*RA*	
1	78/39	4.72	2.57	2h 9m/$178.02

2	74/37	5.80	2.97	2h 50m/$234.50

3	78/39	4.98	3.00	2h/$165.60

4	74/37	5.92	3.18	2h 43m/$224.90

5	78/39	4.05	3.57	28m/$38.64

6	74/37	4.50	2.35	2h 9m/$178.02

7	78/39	7.48	4.05	3h 27m/$285.66

8	74/37	3.93	3.13	47m/$64.86

**Table 6:** Comparison of IM and RA for a matched cohort of eight patients.

Measure	IM	RA	P-value
Average Plan Time (minutes)	74.9	76.6	0.792

Average MUs (SD) (Units)	1813.9 (SD = 159.1)	590.2 (SD = 67.1)	<0.001

Average Treatment Time (SD)* (hours)	5.2 (SD = 1.2)	3.1 (SD = 0.5)	0.001

Average Treatment Staff Cost per Patient*	$ 489.91	$ 315.66	0.001

*Total time calculated for all fractions

## Discussion

In our audit, 30 prostate cancer patients treated radically with RA by one radiation oncologist were found to be treated with acceptable toxicity. In re-planning eight prostate cancer patients treated with definitive external beam, 3D was found to be incapable of covering a more modern radiotherapy volume even at the higher tolerances allowed with that technique. It is therefore definitely time for Australian radiotherapy to move on from 3D.

Modern radiotherapy volumes can be treated via conventional IM and RA, even at the more exacting dose constraints demanded by our local guidelines. There was no difference in planning times between these techniques. However, RA was significantly superior in terms of decreased monitor units and therefore safety as least as far as second malignancy risk is concerned [2,3]. RA also had a decreased treatment time, and so decreased treating staff time, and therefore costs. The average total beam time per radiotherapy course with IM was over two hours more than with RA. There was an average saving of treating staff costs for each patient of $AUD174 with RA over IM. RA cost only 64% of the treating staff cost of IM. The real saving is greater, as only the treating staff costs were computed, not the extra cost implicit in the extra time needed for keeping the department open with administration and nursing staff etc, nor the extra capital costs for more buildings and machines that would be necessary to treat the same number of patients in a timely fashion. For interest we looked at a group of prostate cancer patients treated for the same indications by the same radiation oncologist and with the same machine with 3D before it was commissioned for RA. We found that the there was no difference in the average total beam time between the RA and the 3D groups (p = .885). RA then compares favorably with 3D from a logistical viewpoint with similar treatment times and therefore costs. The superior dosimetry, and monitor unit savings makes it the preferred technique. RA overall combines the superior dosimetry of IM, the logistics of 3D, and yet with a better safety profile.

RA efficiency means even more. Patients are on the hard accelerator bed for less time, so patient comfort is improved. There is less time for internal organ intrafraction motion. Less treatment time per patient also leads to better clinical flow. More indications for radiotherapy can be treated with this new technique. Even palliative regimes can now access VMAT radiotherapy e.g. whole brain radiotherapy with simultaneous integrated boost and hippocampal sparing [7]. Our conclusion is that RA was superior to the other modalities, even conventional IM.

The finding of superiority of RA in this study is important in the Australian context. RA is just one VMAT technology now available. Australian centres have been plagued by skilled staff shortages and waiting lists [8,9]. VMAT can contribute to solving these problems as well as update our treatment complexity to the level expected of a developed country.

## Conclusions

Thirty prostate cancer patients treated radically by one radiation oncologist with Rapid Arc^® ^(RA), a type of volumetric modulated arc therapy (VMAT) were treated with acceptable toxicity. When eight of these patients were re-planned, three dimensional conformal techniques (3D) was not capable of covering the volumes needed without exceeding local guidelines for toxicity. RA was significantly superior to conventional intensity modulated radiotherapy (IM) with more efficient total treatment times, less monitor units and with no increase in planning times. The average treatment staff cost per patient course of radiotherapy was decreased from $489.91 to $315.66. RA combines the superior dosimetry of IM, the logistics of 3D, and yet with a better safety profile.

## List of Abbreviations

VMAT: Volumetric Modulated Arc Therapy; RA: RapidArc; IM: Intensity modulated radiotherapy; 3D: Three-dimensional conformal radiotherapy; MU: Monitor Unit; Gy: Gray (unit of radiation); RTOG: Radiation Therapy Oncology Group; IGRT: Image guided radiotherapy.

## Competing interests

The authors declare that they have no competing interests.

## Authors' contributions

GBF conceived the study, created the study design and drafted the manuscript. DN and GL participated in the data collection, coordination of the study and conduct of the study experiment. LEH performed the statistical analysis and assisted in the drafting the manuscript. NB participated in the conduct of the experiment. All authors have read and approved the final manuscript.
